# Male age and female mate choice in a synchronizing katydid

**DOI:** 10.1007/s00359-015-1012-9

**Published:** 2015-05-10

**Authors:** M. Hartbauer, M. E. Siegert, H. Römer

**Affiliations:** Institute of Zoology, Karl-Franzens University Graz, Universitätsplatz 2, 8010 Graz, Austria

**Keywords:** Mate choice, Signal timing, Chorusing, Insect, Asymmetric preference

## Abstract

In acoustically communicating species, females often evaluate the frequency content, signal duration and the temporal signal pattern to gain information about the age of the signaller. This is different in the synchronizing bush cricket *Mecopoda elongata* where females select males on the basis of relative signal timing in duets. In a longitudinal approach, we recorded songs of *M. elongata* males produced 2 weeks (young male) and 9 weeks (old male) after their ultimate moult. Signal timing of both age categories was studied in acoustic interactions, and female preference was investigated in choice situations. Young male chirps were significantly shorter and contained less energy compared to “old chirps”. In mixed-age duets younger males timed their chirps as leader significantly more often. Females preferred the young male chirp when broadcast as leader over the old male chirp, but choice was random when the old male chirp was leader. This choice asymmetry was abolished after reducing the duration of the “old chirp”. Results were mirrored in response of a bilateral pair of auditory neurons, where the asymmetry in spike count and first-spike latency correlated with behaviour. We suggest that older males may compensate their disadvantage in a more complex chorus situation.

**Electronic supplementary material:**

The online version of this article (doi:10.1007/s00359-015-1012-9) contains supplementary material, which is available to authorized users.

## Introduction

In many organisms, sexual selection primarily depends on the variability of signal traits that are associated with the sender’s quality (Maynard 1976; Andersson 1994; Jennions and Petrie 1997). In acoustically communicating insects, signal traits often change with the age of the signaler (e.g. Simmons and Zuk 1992; Ritchie et al. 1995; Simmons 1995; Lehmann and Lehmann 2008; Verburgt et al. 2011) and females may gain fitness benefits by evaluating age-dependent signal traits (Simmons 1986, 1987). Theory about mate choice predicts choice for older males as mates because they likely carry fewer deleterious inherited mutations (Manning 1985) and have proven the ability to survive (Trivers 1972; Halliday 1978; Kokko and Lindstrom 1996). Brooks and Kemp (2001) reviewed the evidence for male age as an indicator of quality and emphasized the importance of life-history for sexual selection. However, the preference for older males in insects is not consistent (Zuk 1987; Galliart and Shaw 1991; Ciceran et al. 1994; Simmons 1995; Lehmann and Lehmann 2008). In a viability-based simulation model, Beck and Powell (2000) demonstrate that in mating systems where juvenile survival is relatively low, females should prefer younger and intermediate-age males (for further arguments see Hansen and Price 1995).

Females selecting among males by sound evaluate signal intensity, complexity or persistence of a male’s signal to gain information about the quality of the sender (Thornhill and Alcock 1983). Therefore, an older male calling for longer periods of time with fewer interruptions may increase its mating success compared to a younger, more hesitantly signalling male (but see Kokko 1997). However, the songs of younger *G. bimaculatus* males contain more energy because of a higher syllable duration which makes songs more attractive for females (Verburgt et al. 2011). A similar female preference for the songs of young males was found in *Tettioniga viridissima* and *Ephippiger ephippiger* (Jatho et al. 1994) where the duration of syllables in the song of young males is higher and the syllable pattern lacks signs of wear of the stridulatory apparatus, such as broken pegs (Ritchie et al. 1995). However, in the majority of mating systems the signal trait that allows females to discriminate between males of different age is still unknown. For example, in *G. camptestris,G. veltis* and *G. pennsylvanicus* females preferentially oriented towards older males within experimental populations (Zuk 1987; Ciceran et al. 1994; Simmons 1995).

In the katydids *Poecilimon zimmeri* and *Amblycorypha parvipenni*s females prefer older males in a choice situation which leads to direct fitness benefits due to the fact that older males are heavier and deliver larger spermatophores compared to young ones (Galliart and Shaw 1991; Lehmann and Lehmann 2008). However, age-based mate choice is more interesting in those mating systems where males only deliver small spermatophores and do only contribute to offspring survival through their genes. In this case, the basis for mate choice often remains obscure and its adaptive significance has to be questioned (Arnold 1983; Trivers 1985). In the tropical bush cricket species *Mecopoda elongata* males only deliver very small spermatophores (~0.4 % of body weight) and females select males in a choice situation on the basis of relative signal timing rather than by evaluating other signal properties (Fertschai et al. 2007). Therefore, it remains to be tested whether or not females are able to discriminate between males of different age solely by evaluating periodic signals produced with a small, but consistent time delay of a few tens of milliseconds. Here, we investigated to what extent calling songs of *M. elongata* change with male age and if age influences signal timing in acoustic interactions. We also studied the ability of females to select a male of an age category in a choice situation. Finally, we aimed to find neuronal correlates of mate choice in a neuroethological approach.

## Materials and methods

### Insects

All experiments were performed with *Mecopoda elongata* (Orthoptera, Tettigoniidae; Mecopodinae) taken from an Institute colony. The colony was established with individuals originally collected in the Malayan rainforest close to the field station Ulu Gombak near Kuala Lumpur. Males of this species generate calling songs identical with “species S” described by Sismondo (1990), consisting of chirps repeated at a regular period (average chirp period (CP) = 2.0 s; Hartbauer et al. 2005). Insects were reared at a 12:12 light:dark cycle at a temperature of 27 °C and 70 % humidity. They were fed ad libitum with fresh lettuce, apple slices, oat flakes and fish food. Males usually start singing after about 2 weeks after their final moult, which is the time when they start producing fertile spermatophores.

### Analysis of acoustic signals of young and old males

We recorded the calling songs of 22 males of different age in a longitudinal approach. Males were physically isolated from each other in plastic containers (dimension 20 × 30 cm) before their last moult. The songs of individual males were recorded early in their adult life (14 days after final moult; young males) and after 63 days (old males). In captivity, males may live as long as 6 months or more, but we decided to restrict the analysis of old male signals to an age of nine weeks, which appears to be a more realistic life time in nature due to strong predation on these insects in the tropical rainforest (Lang and Römer 2008). All behavioural experiments were conducted in complete darkness in the subjective night of this nocturnal insect. Several song parameters and the total time spent calling were subsequently analysed. Since the singing position of males strongly influenced the recorded signal amplitude, it was not possible to directly compare the chirp amplitude produced by males of the two age categories. However, the duration of syllables gives some hints about signal amplitude due to a correlation with the amplitude of syllables (see Hartbauer et al. 2012). Chirps were recorded within the first 10 h of the dark cycle using a tiepin microphone, digitized at a sampling rate of 32 kHz. Although the low sampling rate does not allow analysing the full spectral bandwidth, it was sufficient for gross temporal analysis. Fine temporal and spectral calling song parameters were evaluated on the basis of microphone recordings performed with a 1/4″ free-field condenser microphone (type 40BE with type 26AC, G.R.A.S. Sound and Vibration A/S, Holte, Denmark) connected to a power module (12AK, G.R.A.S. Sound & Vibration A/S, Holte, Denmark). A/D conversion rate of sound signals was 192 kHz and achieved with a firewire sound card (Edirol FA-101, Roland Inc., Tokyo, Japan).

### Data evaluation of solo singing males

25 chirps from the one-third of the initial song bout provided the basis for the evaluation of five signal parameters: chirp duration, intrinsic CP, duty cycle, integral of chirp envelope, signal energy. Prior to signal evaluation all sound recordings passed a digital high-pass filter with a cutoff frequency of 500 Hz to remove background noise unrelated to calling songs. Additionally, the peak signal amplitude was normalized to −0.1 dB full scale in the sound editing software CoolEdit Pro (Syntrillium Software, Phoenix, AZ, USA). Evaluation of the average chirp duration and the intrinsic chirp period (CP) was performed using a custom-written Spike 2 script (v5.2.1, Cambridge Electronic Design, Cambridge, UK). This script also evaluated the duty cycle of calling songs on the basis of chirp duration and CP. The integral of the envelope of the average waveform of 25 chirps and its signal energy were evaluated in MATLAB R2011b (The MathWorks Inc., Natick, MA, USA) after z-transformation. Sonograms of sound signals were computed in Audacity 2.0 using a fast Fourier transformation (FFT) based on 512 points and application of a Hanning window filter.

### Male duets

The influence of age on the ability of males to time their signals either as leader or follower was studied in 20 male duets inside an anechoic room (dimension 220 × 280 × 200 cm). One young and one old male randomly selected from the available pool of males were separated by 100 cm inside metallic wire mesh tubes (diameter 7 cm; length 25 cm). The acoustic interaction of the pair of males was recorded with two tiepin microphones positioned close to each male. Recordings were restricted to the first 10 h of the dark cycle.

### Data evaluation of males interacting in a duet

The time spent calling and number of bouts was manually evaluated in CoolEdit Pro. Leader–follower relationships of males interacting in a duet were analysed in a custom-written Spike 2 script after setting a manual threshold for automatic signal detection. A minimum time lag of 50 ms between the onsets of chirps was chosen to discriminate leader from follower signals, since this time was found to be just sufficient to bias mate choice in two-choice experiments in favour of the leader (unpublished results). The script evaluating leader and follower relations in duets was executed a second time after setting the detection threshold to a higher value, which would mimic a situation in which a receiver detects only syllables of medium and higher amplitude.

### Female choice experiments

The attractiveness of songs typical of a young and old male was studied in no-choice and two-choice experiments. Model songs consisted of a chirp repeated at a CP of two seconds. These chirps had a duration and syllable number typical for the average chirp of each age category (Table 1). The frequency composition of chirps used to model calling songs was not different (see spectra shown in Fig. S1).

**Table 1:** Parameters describing the song and chirps produced by 23 males of different age

Weeks	2		9		Paired *t* test
	Mean	SD	Mean	SD	*P*
Total song duration (s)	2287	2378	4632	2006	**0.001**
Number of bouts	5.0	3.0	6.5	2.6	0.022
Chirp duration (ms)	235	24	260	28	**0.001**
Duty cycle (%)	11.99	1.13	13.00	1.33	**0.001**
Intrinsic chirp period (s)	1.97	0.15	2.01	0.20	0.225
Integral of chirp envelope (t × rel.V)	55.41	7.35	61.21	8.25	**0.001**
Signal energy (mJ/m^2^)	0.45	0.19	0.65	0.33	**0.005**

Bold letters refer to *p*-values smaller than 0.05

Phonotactic experiments were conducted in an acoustically isolated, temperature-controlled chamber. In no-choice trials a song model of a young or aged male was broadcast through a single speaker positioned at a distance of 204 cm from the release site of females. In this experiment the time females needed to approach the speaker was evaluated. In two-choice tests females were given the choice between two speakers broadcasting the young and old male chirp in a leader/follower relationship with a time lag of 70 ms. This time lag is sufficient to bias female choice in favour of the leader of identical signals (Hartbauer et al. 2014). Speakers were positioned 155 cm distant to each other and 210 cm apart from the release point of females, resulting in a stimulus angle of 43.3°. Female walking paths were monitored via a top view infrared camera (CB-38075, GKB, Taichung, Taiwan). Camera frames were digitized with a sampling rate of 30 frames per minute using a video frame grabber card (Video Extreme, PixelSmart, Lewiston, NY, USA). A speaker was regarded as selected by a female if she entered an invisible circle with a diameter of 30 cm surrounding each speaker. From trial to trial, leader and follower signals were exchanged between speakers to eliminate possible influences arising from speaker characteristics and/or female handedness. Fifteen females were tested in two-choice experiments and twelve females in an additional control experiment performed with a shorter “old chirp”. This method leads to data replication which was taken into account in the generalized binomial mixed model (GBMM) used to test for a statistical significant influence of playback signals on female choice (R studio). In the no-choice experiment 10 females were tested three times in each stimulus situation and the time spent approaching the speaker was averaged on an individual basis. Females were isolated from the breed and from males before their ultimate moult. Behavioural experiments were not conducted within 21 days thereafter.

### Neurophysiology

In a neurophysiological approach we studied the representation of signals produced by young and old males in the activity of an afferent auditory neuron with T-shaped morphology (TN1; Suga and Katsuki 1961; McKay 1969). The large axon diameter and its lateral position in the connectives allows to record extracellular activity from both side-homologous TN1 neurons simultaneously with a pair of hook electrodes (Rheinlaender and Römer 1980). In *M. elongata* TN1 encodes the syllable pattern of conspecific signals in a rather robust manner (Siegert et al. 2011). For further methodical details see Siegert et al. (2011). The neuronal preparation was placed in an anechoic chamber with two speakers broadcasting signals that were used in the female choice experiment from a distance of 30 cm. TN1 activity was amplified with an extracellular biosignal amplifier connected to a headstage (EXT-02F/1, npi, Tamm, Germany) and digitized using an A/D board operating at a sampling rate of 40 kHz (PowerLab/4SP, AD Instruments, Spechbach, Germany). The action potential activity of both TN1 neurons was stored in Chart (v5.5.6, AD Instruments, Spechbach, Germany) for later analysis.

### Acoustic playback

Signals used as playback stimuli were originally recorded from isolated singing males inside an incubator at 27 °C. The inner surface of the incubator was covered with sound dampening material. Songs were recorded at a distance of 30 cm with a ¼″ free-field condenser microphone (type 40BE, G.R.A.S. Sound & Vibration A/S, Holte, Denmark) connected to a preamplifier (type 26AC, G.R.A.S. Sound & Vibration A/S, Holte, Denmark) and a power module (12AK, G.R.A.S. Sound & Vibration A/S, Holte, Denmark). Signals were A/D converted with a sampling rate of 192 kHz using a firewire sound card (Edirol FA-101, Roland Inc., Tokyo, Japan) that was operated by a sound editing software (CoolEdit Pro 2.0). The same sound card was also used for the playback of signals in female choice experiments. Playback signals were amplified by a stereo amplifier (NAD 214, NAD Electronics, Pickering, ON, Canada) and broadcast through leaf tweeters (EAS-10TH400A, Technics, Kadoma, Japan) after passing a two-channel signal attenuator (PA-5, Tucker Davis Inc., Alachua, FL, USA). The three last syllables of high amplitude of playback signals were calibrated to 65 dB relative to 20 µPa at the release site of females by calculating their root mean square amplitude with a custom-written MATLAB script (The MathWorks Inc., Version R2011b, Natick, MA, USA).

### Statistical tests

With the exception of data gathered from female choice experiments, all statistical analyses were performed in Sigmaplot (version 12.0, Systat Software Inc., Chicago, IL, USA). Data sets were evaluated for linearity before performing parametric tests. A significant preference of females for a song model in two-choice situations was investigated in RStudio (Version: 096.230, R version: 2.13.1) by application of a generalized binomial mixed model (GBMM) fitted by Laplace approximation. In this model female ID acted as a random intercept.

## Results

### Signal properties related to male age

**Fig. 1:**
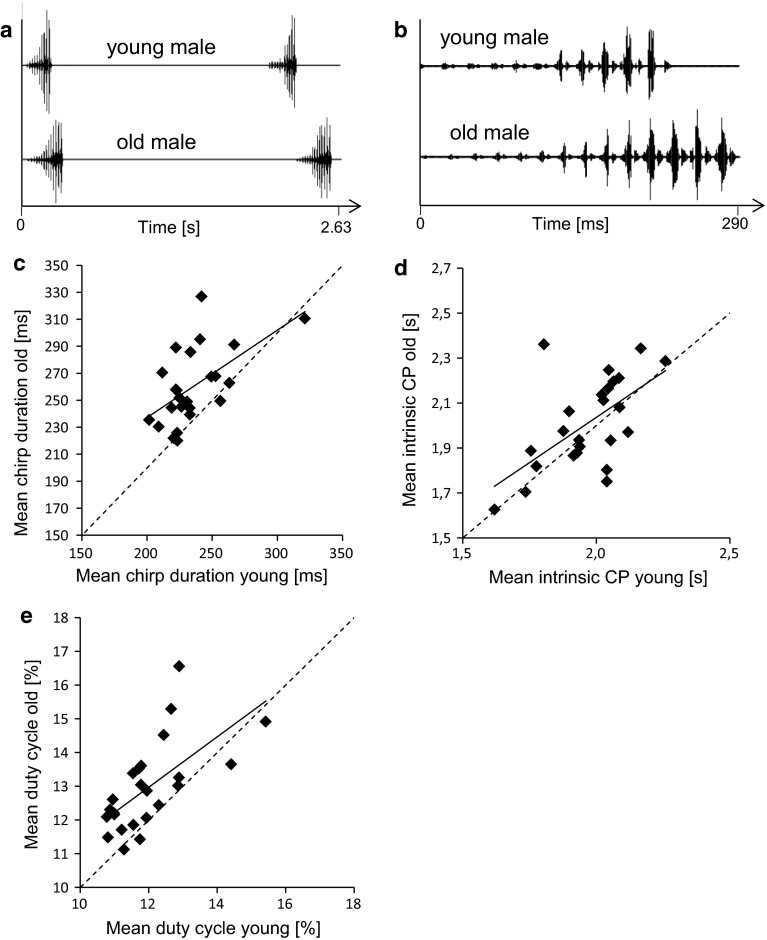
Song and chirp parameters of young and old males. **a** Short section of the calling song of the same male recorded either 2 weeks (young) or nine weeks (old) after his ultimate moult. **b** The chirp of each age class shown at higher temporal resolution. **c**–**e** Scatterplots of the mean chirp duration, intrinsic chirp period and duty cycle of the calling songs of 24 males of different age

Correlation analysis of morphological parameters with various song parameters describing solo songs of young males revealed that neither pronotum width nor femur length is correlated with the average signal period, signal duration or song duty cycle (*p* > 0.05, Spearman rank order correlation, 22 males). However, old males spent significantly longer time singing compared to young males (Table 1). This difference in signalling effort is due to significantly longer song bouts of old males because the total number of song bouts was similar in both age categories. The chirp duration of old males is often higher compared to young males (see example in Fig. 1a, b). Solo songs of 13 of a total of 24 males were characterised by a significantly higher average signal duration when singing as an old male compared to a young male (Table 1; Fig. 1c). The average intrinsic CP was similar in both age groups (Table 1) and the comparison on an individual basis showed that only three males changed their intrinsic CP by more than 150 ms (Fig. 1d). However, there is a slight tendency of males to extend intrinsic CP as they age. 13 of 24 males exhibited a significantly higher duty cycle when old compared to young (Fig. 1e). Furthermore, the average duty cycle of old males was significantly higher compared to young males (Table 1).

**Fig. 2:**
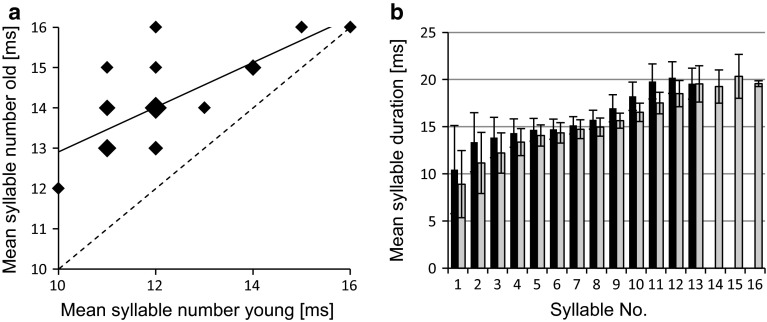
**a** *Scatterplot* of the average number of syllables of chirps produced either as young (*x*-axis) or old male (*y*-axis). The size of *diamonds* indicates the number of overlapping data points. **b** Average duration of syllables of young (*black bars*) and old males (*grey bars*) (*N* = 20 chirps of 24 males). *Error bars* SD

Fine temporal analysis of the chirps showed that old males generate a higher number of syllables (Fig. 2a) but the duration of syllables increased faster from syllable to syllable in young males (Fig. 2b, black bars). Chirps of old males consist of a higher number of long-lasting syllables, which suggests that their chirps are louder than the chirps of young males. Chirps of old males contained significantly more energy and exhibited a higher area of the waveform envelop compared to young males (Table 1). By contrast, there was no consistent pattern of differences in the frequency content of chirps between the two age groups (data not shown). A detailed analysis of the duration of loud hemisyllables and the amplitude modulation of loud and soft hemisyllables are shown in Fig. S2.

### Male duets

When young and old males interacted acoustically in 20 duets, the time spent singing was not significantly different between both age categories (Table 2). Older males were slightly more motivated to initiate song bouts and initiated 4.7 of 7.6 song bouts. Similar to the solo singing situation, the average signal duration of aged males was significantly higher in acoustic duets with younger males (*p* < 0.001, Mann–Whitney rank sum test, *N* = 68 bouts). This also holds true when only syllables of medium to high amplitude were evaluated (Table 2). Young males timed a significantly higher proportion of signals as leader with a time lag of more than 50 ms when interacting with the other age category (young: 36 %, old: 26 %; *p* < 0.001, *z* test, *N* = 20 duets). A similar result was found when only medium and loud syllables were evaluated (young: 27 %, old: 20 %; *p* < 0.05, *z* test, *N* = 20). The average time difference of signal onsets during synchronous acoustic interactions was 72 ± 56 ms and thus very similar compared to the mean time lag separating the onsets of medium to high amplitude syllables (69 ± 159 ms; 20 duets each consisting of 2–4 song bouts).

**Table 2:** Song and signal parameters obtained from 20 duets consisting of a young and aged male

Weeks	2		9		*N*	*P*
Duet	Mean	SD	Mean	SD		
Total song duration (s)	5078	2972	5579	2826	39	>0.05
Mean chirp duration (ms)	226	29	253	28	68 bouts	<**0.001** ^a^
Mean duration of loud syllables (ms)	68	28	93	35	68 bouts	<**0.001** ^a^
Proportion of leading chirps (%)	35.5	23.5	25.8	17.9	68 bouts	<**0.001** ^**b**^
Proportion of loud leading syllables (%)	26.9	23.6	19.8	15.9	68 bouts	<**0.05** ^**b**^

Bold letters refer to *p*-values smaller than 0.05

^a^Mann–Whitney rank sum test

^b^ *z* test

### Female choice

**Fig. 3:**
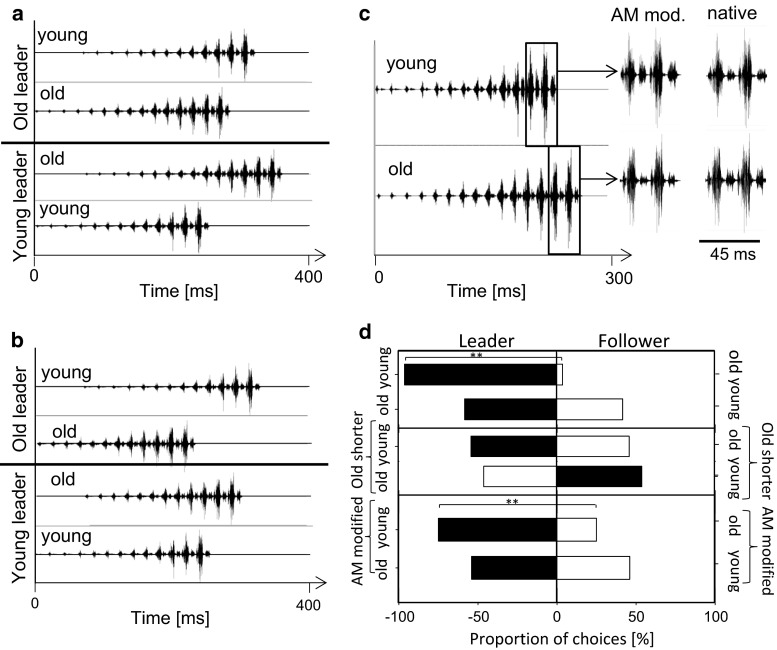
Female choice experiments. **a** Signal timing of the chirps presented either as leader or follower in mate choice and neurophysiological experiments. A time lag of 70 ms separates chirp onsets. **b** Same as in (**a)**, but with a shorter “old chirp”. **c** The chirp of the young male had a higher AM of syllables compared to the old chirp (see details *right*). The influence of AM differences on female choice was tested by manipulating the amplitude of soft syllables. **d** Relative proportion of preference when either the chirp of a young male was broadcast as leader and the chirp of an old male as follower or vice versa (*upper bars*). Female preference between songs consisting of the shorter old chirp and AM manipulated chirps (*lower bars*)

A previous study had shown that females given the choice between identical conspecific signals broadcast from different directions with a small temporal delay preferably oriented towards the source of the leader signal (Fertschai et al. 2007). Those males exhibiting faster intrinsic chirp rates are more likely to time signals as leader in a duet (Hartbauer et al. 2005) and a small chorus consisting of four males (Hartbauer et al. 2014). In the current study females approached the speaker broadcasting the model chirp of the young male significantly faster in no-choice tests compared to the old male chirp (*p* < 0.05, paired *t* test; 73.6 ± 42.1 s vs. 111.0 ± 86.5 s; *N* = 10 females). In two-choice tests, young and old male chirps were either broadcast as leader or follower (Fig. 3a). When the young male chirp was broadcast as leader, females showed a strong preference for the young male chirp (*p* < 0.001, GBMM, *N* = 15). By contrast, female choice was random when the old male chirp was broadcast as leader (*p* > 0.05, GBMM, *N* = 15 females, Fig. 3d). This result may be due to differences in the modulation depth of hemisyllables between both song models (young chirp: 78 %; old chirp: 65 %). We, therefore, manipulated the amplitude pattern of loud and soft hemisyllables in a way that made the young chirp look old and vice versa (Fig. 3c). This was achieved by increasing the amplitude of soft syllables in the young chirp and attenuation of soft hemisyllables in the old chirp. However, this manipulation neither affected the preference of females for the young chirp when presented as leader nor did it affect the lack of preference when the old chirp was broadcast as leader (Fig. 3d). Another signal property that may affect the preference of females given the choice between young and old male chirps is signal duration since a longer signal of the old male causes a delay in the timing of loud syllables (82 ms), when the “young chirp” is broadcast as leader with a temporal advantage of 70 ms (Fig. 3a). In a control experiment we removed four soft syllables of the “old chirp” to reduce signal duration to 232 ms. This manipulation removed the asymmetry in the timing of loud syllables in the “young leader” situation but created an asymmetry in the “old leader” situation (Fig. 3b). Twelve females given the choice between a shorter “old chirp” and a native “young chirp” presented either as leader or follower did not show a preference for any signal (*p* > 0.05, GBMM, *N* = 12 females, Fig. 3d). Notably, the motivation to approach any speaker in these control experiments was very low (36 runs of 133 trials).

### Neurophysiology

**Fig. 4:**
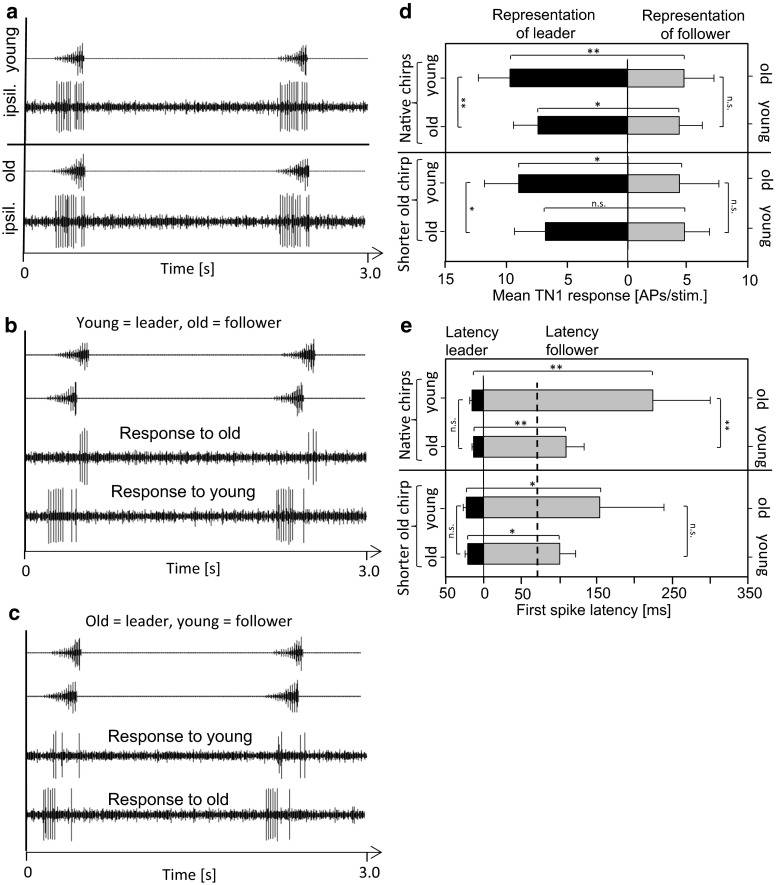
Neuronal representation of acoustic signals of young and old males in the bilateral pair of TN1-neurons. **a** Example of TN1 response to the ipsilateral playback of either the young (*upper panel*) or the old chirp (*lower panel*). **b**, **c** Example of TN1 responses to a simultaneous broadcast of the young and old chirp from opposite sides, with either the young (**b**) or old male chirp (**c**) broadcast as leader with a time lag of 70 ms. (**d**) Average bilateral TN1 response to the young and old chirp broadcast with a time lag of 70 ms (*N* = 10 individuals). The average bilateral TN1 response to a shorter old chirp (234 ms) in the same L/F situation (**d**, *lower panel*, *N* = 6). **e** Average first-spike latency of the bilateral TN1 response to the young and old chirp either broadcast as leader or follower with a time lag of 70 ms (indicated by the *vertical dashed line*). Note a strong reduction of TN1 spike latency on the follower side after reducing the duration of the old chirp to 232 ms

The results of two-choice experiments may have a rather “simple” neuronal basis, if the activity in the afferent auditory pathway in response to chirps of young and old males is different. We tested this prediction in bilateral recordings of the pair of TN1-neurons, which respond to conspecific chirps quite well (Siegert et al. 2011). Both chirp models activate the TN1-neuron equally strong, when broadcast without a competitive chirp (see example in Fig. 4a). On average young chirps elicited 11.3 ± 3.1 spikes and old chirps 10.5 ± 3.5 spikes per signal presentation (*N* = 10 preparations). In a leader/follower situation with a time lag of 70 ms there is a strong asymmetry in the responses of both neurons, with a stronger response to leader chirps (see example in Fig. 4b, c). In this situation, leader chirps elicited on average about 10 APs per L/F stimulus whereas the follower chirp elicited only about 4 APs (Fig. 4d). However, when the chirp of a young male was broadcast as leader the average asymmetry of response elicited in both neurons was significantly stronger in favour of the young male chirp as compared to the leadership of the old male chirp (Fig. 4d). This was mainly due to a stronger response in the neuron on the leader side (see example in Fig. 4b).

The young male leader advantage in the neuronal representation reported above may result from the difference in the signal duration of both signals (young chirp: 258 ms; old chirp: 292 ms). As a consequence, the time lag of the onsets of syllables of medium amplitude is 82 ms when the “young chirp” is presented as leader, but only 40 ms with the old chirp as leader (Fig. 3a). To compensate for this effect, the duration of the “old chirp” was reduced to 232 ms by removing four initial syllables of low amplitude (see the same signal paradigm for choice experiments above). Although this manipulation reduced the time difference between the onsets of loud syllables to 28 ms with the “young chirp” as leader, the average neuronal response of both TN1 neurons was not different compared to a situation with the original “old chirp” (Fig. 4d). Evaluation of the first-spike latency of the bilateral TN1 response revealed that spike latency on the follower side exceeded the stimulus time lag of 70 ms by far (dashed line in Fig. 4e). Interestingly, the average latency of TN1 response to the follower signal was much higher when the “old chirp” was follower compared to broadcasting the “young chirp” as follower (224 ms vs. 109 ms; Fig. 4e). Shortening of the “old chirp” by withdrawing four soft syllables caused a strong reduction of first-spike latency on the follower side.

## Discussion

Changes in signal traits during the adult life of males can potentially be used by females for mate choice decisions. Such changes may come about in acoustic insects either through extensive use of the stridulatory apparatus modifying fine temporal or spectral properties (Hartley and Stephen 1989; Ritchie et al. 1995), or changes in the neural substrate for sound production so that the temporal pattern or duration of song elements vary over lifetime (Jatho et al. 1994; Richtie et al. 1995; Verburgt et al. 2011). However, the precise signal trait that allows females to discriminate between males of different age is often unknown ( Zuk 1987; Galliart and Shaw 1991; Ciceran et al. 1994; Simmons 1995; Lehmann and Lehmann 2008).

In the synchronizing katydid *M. elongata* the situation is more complicated since a major determinant for female mate choice is not the single male acoustic signal, but the fine-scale temporal relationship with that of other males calling in imperfect synchrony (Fertschai et al. 2007; Hartbauer et al. 2014). In our study, we, therefore, followed changes in the acoustic properties of male chirps over lifetime both singing in isolation, and in addition in song interactions between each age class.

The average intrinsic signal period of young and old males is similar, but within male comparison revealed that the signal period of males slightly increases as they age (Fig. 1d). Given a higher likelihood of intrinsically faster signalling males to time signals as leader (Hartbauer et al. 2005), we expected that young males become leader more often compared to old males in acoustic duets. Indeed, mixed-age duets revealed that young males time a significantly higher proportion of signals as leader compared to old males (Table 2). Instead, older males increased the duration of their song bouts as well as duty cycle by producing long-lasting chirps. Additionally, older chirps also have a higher energy content, mainly achieved by increasing the number of loud hemisyllables (Fig. 2b). This is consistent with previous work on other species where older males increase their calling effort (Latimer and Schatral 1986; Williams 1966; Allen 2000). Also, old males sang for longer periods of time and more often initiated song bouts in male acoustic interactions.

However, the strongest age-related effect should be expected through changes in the way males synchronize their chirps with other males, since females exert a strong selection on male signalling by preferentially choosing the leader (Fertschai et al. 2007; Hartbauer et al. 2014). To our surprise, we found an asymmetry in the effect of the leader role, depending on whether the chirp of the young or old male was the leader (Fig. 3d): the preference was stronger with the young male chirp, irrespective of differences in the AM of playback signals because the preference of the “young chirp” in two-choice tests is not related to differences in the amplitude pattern of hemisyllables (Fig. 3c). The preference was corroborated in the no-choice situation where females covered the distance to the speaker in a shorter period of time when the young song was broadcast. Thus, contrary to the expectation that a longer duration signal should be favoured by females both in no-choice tests and in synchronous interactions, this was not the case.

A possible explanation for this unexpected result in the choice situation comes from the timing of loud syllables when either the young or the old chirp is broadcast as follower. Due to a higher number of soft syllables typical for chirps of older males, loud syllables are delayed when the “old chirp” is lagging 70 ms behind the leader (Fig. 3a). Our control experiments performed with the shorter “old chirp” lacking four soft syllables demonstrate that manipulation of signal duration was sufficient to compensate the advantage of the “young chirp” broadcast as leader. At the same time the “shorter old” chirp presented as leader did not lead to a higher attractiveness of this signal despite a similar time delay between loud syllables of chirp alternatives (Fig. 3b). Therefore, females seem to evaluate the timing of loud syllables for mate choice decisions. Indeed, follower signals produced by males interacting in duets are characterised by a smaller number of soft syllables (Hartbauer et al. 2012), which suggests that males avoid the production of late loud syllables by dynamically reducing the number of soft syllables. However, duets of males in the two age categories revealed that the timing of signal onset as well as the onset of loud syllables was about 70 ms, which is sufficient to bias mate choice towards the leader (Hartbauer et al. 2014). Thus, females given the choice between a young and old male likely prefer young males with a higher proportion of leader signals (Table 2). However, there is evidence that signal duration plays an important role for mate choice when the choice situation is more complex (Hartbauer et al. 2014). When females were released within a small chorus of four acoustically interacting males only 40 % of females preferred males producing the majority of leading signals, despite the fact that the leader role is so important in two-choice trials. Rather, the highest preference was observed for males which had a frequent leader role and simultaneously producing longer signals compared to the competing males (Hartbauer et al. 2014). Therefore, it is likely that older males, by producing long-lasting chirps, are at least equally attractive compared to young males in the complex choice situation of a chorus.

We also aimed at studying the potential proximate neuronal mechanism for the observed female preferences, which could be based on asymmetrical responses in bilateral pairs of afferent neurons, when these chirps are produced by spatially separated males (Römer et al. 2002; Siegert et al. 2013). A similar neuronal response to the young and old chirp was found when presented without a competing chirp. Nevertheless, consistent with the behavioural data, the “young chirp” as leader signal elicited a significantly stronger neuronal response asymmetry in the pair of TN1-neurons compared to the situation when the old male chirp was the leader signal. Since the very small spectral differences between both song models are unlikely to account for a stronger response to the young chirp (Fig. S1), we hypothesized that differences in signal duration between chirps produced by young and aged males account for the preference of the young chirp in a choice situation. Although the reduction of signal duration of the old chirp did not change the average response magnitude of both TN1 neurons (Fig. 4d), this manipulation strongly affected first-spike latency on the follower side (Fig. 4e). Therefore, we hypothesize that spike count as well as spike timing differences between both TN1 neurons represent the proximate cue for the preference of females in a choice situation. This assumption gets support from the behavioural experiment where the shorter “old chirp” abolished the preference of the “young chirp” in the choice situation.

## Electronic supplementary material

Supplementary material 1 (PDF 101 kb) Frequency spectrum of chirps used in playback experiments. Solid line: young chirp. Dashed line: old chirp

Supplementary material 2 (PDF 99 kb) Duration and modulation depth of syllables of 24 males recorded after 2 weeks and 9 weeks after the final moult. (a) Average duration of loud hemisyllables of young (black bars) and old chirps (grey bars). (b) Average modulation depth of loud and soft hemisyllables. Data are based on the evaluation of 3 loudest syllables of 4 representative chirps. Asterisks indicate significant differences between both age classes
