# Supplementary figures and images for: Male age and female mate choice in a synchronizing katydid
Source: J Comp Physiol A Neuroethol Sens Neural Behav Physiol. 2015 May 10;201(8):763–72. doi: 10.1007/s00359-015-1012-9 (PMC4511073; doi:10.1007/s00359-015-1012-9)

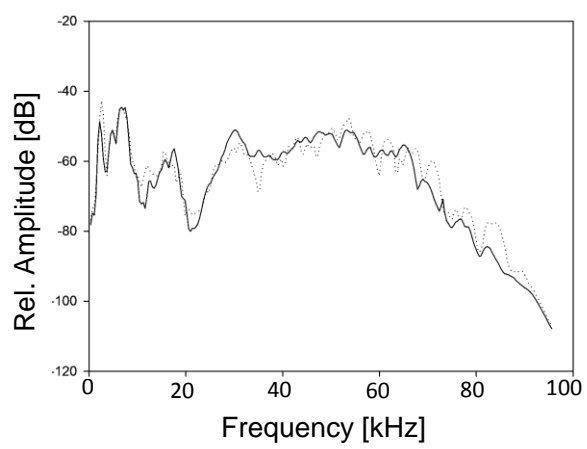

Supplement: Supplementary file 1 — Supplementary material 1 (PDF 101 kb) Frequency spectrum of chirps used in playback experiments. Solid line: young chirp. Dashed line: old chirp [file 359_2015_1012_MOESM1_ESM.pdf]

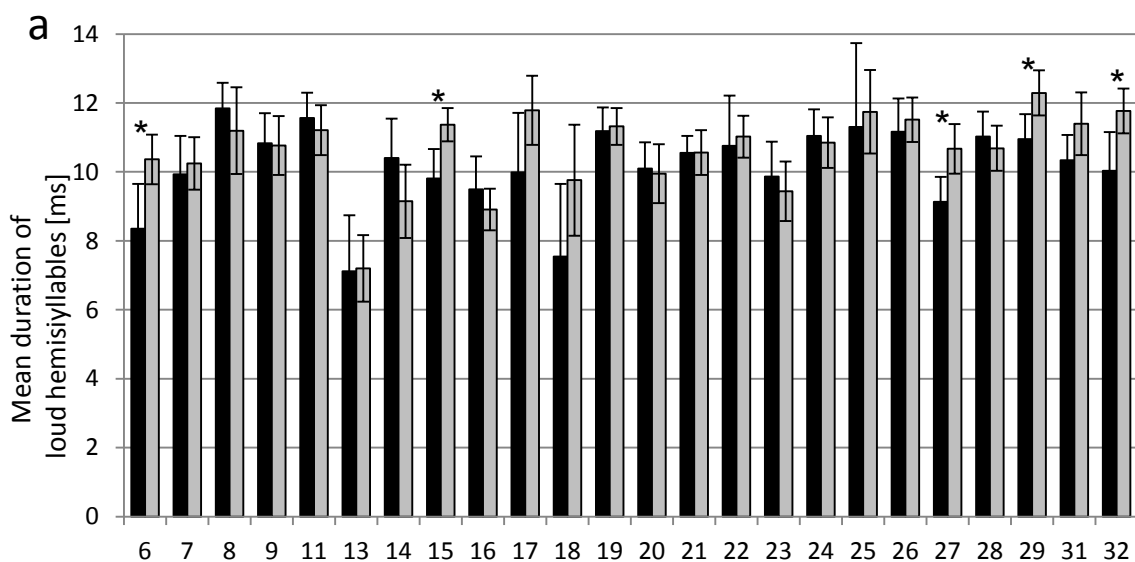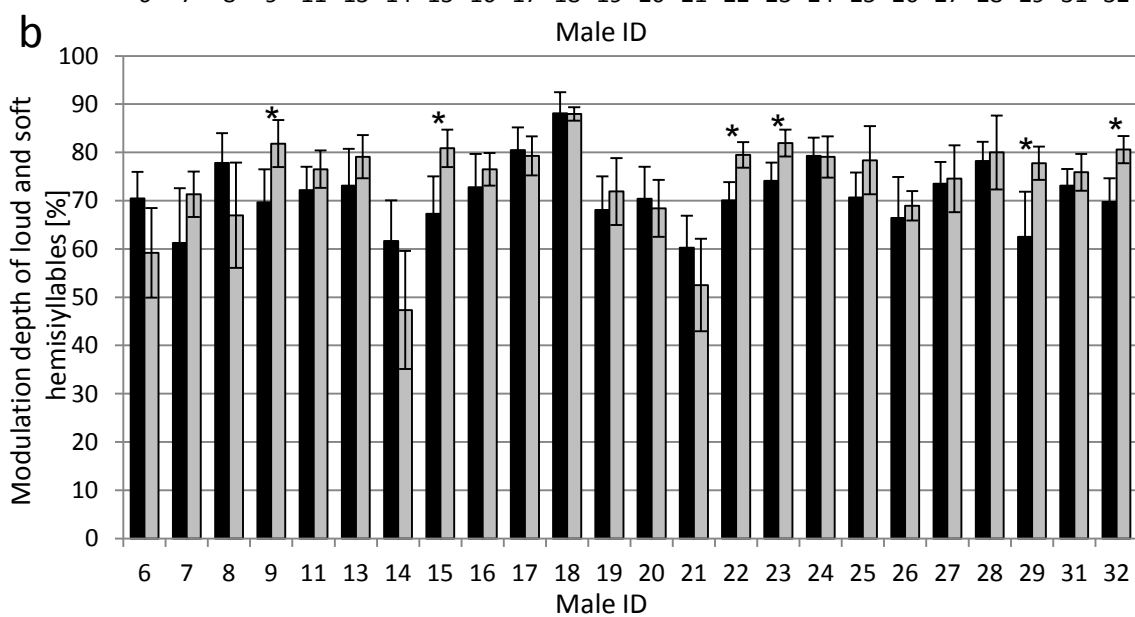

Supplement: Supplementary file 2 — Supplementary material 2 (PDF 99 kb) Duration and modulation depth of syllables of 24 males recorded after 2 weeks and 9 weeks after the final moult. (a) Average duration of loud hemisyllables of young (black bars) and old chirps (grey bars). (b) Average modulation depth of loud and soft hemisyllables. Data are based on the evaluation of 3 loudest syllables of 4 representative chirps. Asterisks indicate significant differences between both age classes [file 359_2015_1012_MOESM2_ESM.pdf]
